# Validation of a Simple HPLC-Based Method for Lysine Quantification for Ruminant Nutrition

**DOI:** 10.3390/molecules26144173

**Published:** 2021-07-09

**Authors:** João Albuquerque, Susana Casal, Rebeca Cruz, Ingrid Van Dorpe, Margarida Rosa Garcez Maia, António José Mira Fonseca, Ana Rita Jordão Cabrita, Ana Rute Neves, Salette Reis

**Affiliations:** 1 LAQV, REQUIMTE, Departamento de Ciências Químicas, Faculdade de Farmácia, Universidade do Porto, Rua Jorge Viterbo Ferreira n.° 228, 4050-313 Porto, Portugal; sucasal@ff.up.pt (S.C.); rebecca.ccruz@gmail.com (R.C.); rutepneves@gmail.com (A.R.N.); shreis@ff.up.pt (S.R.); 2 LAQV, REQUIMTE, ICBAS, Instituto de Ciências Biomédicas Abel Salazar, Universidade do Porto, Rua Jorge Viterbo Ferreira n.° 228, 4050-313 Porto, Portugal; mrmaia@icbas.up.pt (M.R.G.M.); ajfonseca@icbas.up.pt (A.J.M.F.); arcabrita@icbas.up.pt (A.R.J.C.); 3 PREMIX-Especialidades Agrícolas e Pecuárias, Lda, Parque Indústrial II–Neiva, 4935-232 Viana do Castelo, Portugal; invando@premixportugal.com; 4 CQM—Centro de Química da Madeira, Campus da Penteada, Universidade da Madeira, 9020-105 Funchal, Portugal

**Keywords:** high-performance liquid chromatography, lysine quantification, biological samples

## Abstract

Robust and selective quantification methods are required to better analyze feed supplementation effectiveness with specific amino acids. In this work, a reversed-phase high-performance liquid chromatography method with fluorescence detection is proposed and validated for lysine quantification, one of the most limiting amino acids in ruminant nutrition and essential towards milk production. To assess and widen method applicability, different matrices were considered: namely Li_2_CO_3_ buffer (the chosen standard reaction buffer), phosphate buffer solution (to mimic media in cellular studies), and rumen inoculum. The method was validated for all three matrices and found to be selective, accurate (92% ± 2%), and precise at both the inter- and intra-day levels in concentrations up to 225 µM, with detection and quantification limits lower than 1.24 and 4.14 µM, respectively. Sample stability was evaluated when stored at room temperature, 4 °C, and −20 °C, showing consistency for up to 48 h regardless of the matrix. Finally, the developed method was applied in the quantification of lysine on real samples. The results presented indicate that the proposed method can be applied towards free lysine quantification in ruminant feeding studies and potentially be of great benefit to dairy cow nutrition supplementation and optimization.

## 1. Introduction

Lysine (Lys), along with methionine, is often considered the most limiting amino acid (AA) for ruminant production, particularly when considering milk production in dairy cows, mainly due to the AA profile of the currently used corn-based diets [1,2,3,4,5]. Due to the importance of this AA and its natural scarceness, selective and efficient Lys quantification methods are required, able to accurately evaluate this amino acid in feed ingredients and diets, ensuring that adequate amounts of the AA are being fed to the animal, as well as to evaluate it after feeding, to evaluate Lys release in the rumen or even its uptake at cellular level.

Due to Lys’ non-chromogenic nature, a conversion into chromogenic or fluorescence derivatives prior to analysis and quantification is the standard approach [6,7,8,9,10]. Several high-performance liquid chromatography (HPLC) methods already exist for the separation and quantification of AA in biological samples [6,11,12,13,14,15,16]. However, most of these methods are time consuming [6,11,12,16] and some may require expensive derivatization methods [13] or high temperatures [16] which reduce their applicability. Furthermore, most developed methods are designed to quantify multiple AA in specific media and have yet to be optimized for media such as the rumen inoculum, which poses a particular challenge to ruminant nutrition.

The method proposed herein is based on HPLC with fluorescence detection, and was optimized to achieve a fast and selective Lys quantification that could be applied in several biological scenarios regarding studies in ruminant animals. This method was developed to be used in three matrices: (1) Li_2_CO_3_ buffer—chosen as the standard reactionary media [12]; (2) phosphate buffer solution (PBS)—selected to mimic cellular assay conditions; and (3) rumen inoculum—selected for in vivo or ex vivo simulation of the fermentation in this organ. Moreover, sample stability was assessed for up to 96 h of storage at three different storage temperatures, to determine the most suitable way to store samples in the eventuality that an immediate quantification is not possible.

## 2. Results and Discussion

### 2.1. Method Optimization

The chromatographic conditions of the original method [12], developed for the separation of eight dansylated AA, were selected as a working basis. This original method was not tested with more complex media such as those found in animal science applications, with only Li_2_CO_3_ buffer being considered. For these reasons, the chromatographic conditions were optimized for a faster Lys quantification in several biological matrices, due the importance of this AA in dairy cow nutrition.

Dansyl chloride is one of the most widely used derivatization agents in the quantification of AA [11,12,17,18,19,20,21,22], and its derivatives do not suffer from poor stability such as the ones obtained when using o-phthalaldehyde [23]. Although the latter’s derivatization method is simpler and faster [23], the different stability of derivatives was the main reason dansyl chloride was chosen as the derivatizing agent in this work. In short, the derivatizing agent’s concentration was increased 10-fold, to ensure a complete analyte derivatization in complex matrices and concentrated samples. Methylamine was selected to consume the excess of derivatizing agent, as it quickly eluted from the column, even when derivatized, and would not cause overlay with any of the peaks of interest. The initial gradient [12] was altered in order to reduce total run length without compromising the separation of Lys. An internal standard (IS) was added to improve the method, with L-phenylalanine ethyl-ester being the selected substance, due to it not occurring naturally in the rumen, and due to its peak’s proximity with that of Lys, without overlapping it. After this optimization process, the chromatographic method was characterized using the new conditions (Table 1).

The optimization of the chromatographic conditions effectively reduced the total run length from 60 min to 22 min, and Lys retention time from 44 min to 13.5 min, without impairing Lys separation from matrix impurities, and with a dead time (t0) of 1.2 min. Additionally, the method presented retention factors higher than 6 for both analyte and IS (Table 1) which are indicative of a good separation [24]. The resolution between the peaks of the two substances, Lys and IS, was also high (≥1.5), ensuring that they are well separated at the baseline [24].

### 2.2. Method Validation

**Figure 1:**
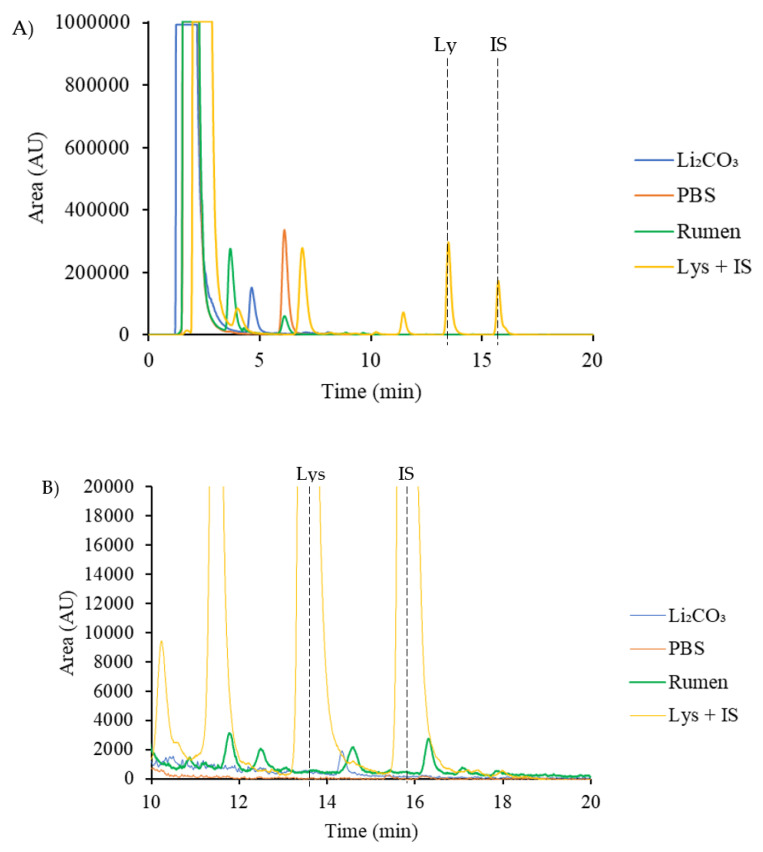
Chromatogram of Li_2_CO_3_ buffer, phosphate buffer solution (PBS), and rumen fluid and lysine (Lys), with internal standard (IS) in Li_2_CO_3_ buffer, showing the entire run length (**A**) and a zoomed in interval, relevant for Lys quantification (**B**).

Selectivity is the methods’ capability to differentiate the analyte from the remaining components of the matrix [25]. The HPLC gradient was previously optimized to achieve a complete baseline separation between dansylated Lys and the IS. Using the optimized gradient, no other peaks were observed near both Lys and IS retention times with retention factors of 6.9 and 8.2, respectively (Table 1), indicating that the method allowed an effective separation of the components in all matrices (Figure 1).

Linearity was evaluated by plotting calibration curves constructed as previously described. The study showed, with the tested chromatographic conditions, a good linearity (R ≥ 0.9996) within the proposed range for all matrices. The range, slopes, and correlation coefficients of plotted regression curves for Li_2_CO_3_ buffer, PBS buffer and rumen inoculum are presented in Table 2. These curves were linear and reproducible in the tested concentration range (1–225 µM for Li_2_CO_3_ and PBS, and 5–225 µM for rumen inoculum). It should be noted that the range for which the rumen inoculum curve was valid was slightly smaller, corresponding to the expected concentration under live assays. 

The limit of detection (LOD) and limit of quantification (LOQ) values (Table 2) were very similar for both buffers and higher for rumen inoculum, as a result of a more complex organic matrix (Figure 1B). Additionally, both LOD and LOQ values determined for the buffers had the same order of magnitude as those presented in the original method [12]. The linearity of both the proposed and the original method was good, as indicated by the high correlation coefficients (Table 2), but a direct comparison could not be performed as the concentration range was not stated in the original method [12]. Despite a direct comparison not being possible, the tested concentration range is in accordance with the literature [6,13,15,16,26].

Accuracy and precision were determined by analyzing a chromatographic control (CC) sample with 25 µM of Lys, with added Lys or as is. This concentration was chosen due to being an intermediate value, when considering a logarithmic scale, within the tested concentration range. Precision depicts how close the values of different replicas are to each other. CC samples with Lys were derivatized immediately before the analysis and the intra- and inter-day relative standard deviation (RSD) values calculated (Table 3). The accuracy of an analytical method relates to how close the rendered values are to the nominal values. 

The results obtained regarding accuracy and precision are similar to the ones obtained using the original method [12], particularly when comparing the values of Li_2_CO_3_ buffer, but also when considering the values of PBS and rumen inoculum, with the exception of findings for intra-day rumen inoculum and inter-day PBS that were considerably higher. Regardless, all values were within the acceptable range according to [27], except for the inter-day RSD for the PBS matrix, indicating that proposed method is accurate and precise when applied to quantify Lys within the tested range.

To determine the optimal sample storage procedure, CC samples of all three matrices were derivatized prior to storage (A), or stored prior to derivatization and derivatized immediately before injection (B). Sample stability was estimated over the course of 96 h and at three temperatures: room temperature (RT), 4 °C, and −20 °C (Table 4). Samples derivatized after storage (B) failed to be stable under any of the tested storage conditions, as well as samples derivatized before storage (A) and stored at −20 °C. In samples that were derivatized prior to storage, the stability varied depending on the matrix tested, with PBS and rumen inoculum presenting the highest stability for up to 96 h if stored at RT or 4 °C, whereas Li_2_CO_3_ only appeared to be stable for 24 h under the tested storage conditions. This overall instability was mainly due to the degradation of the IS, and to a smaller extent to the degradation of Lys, as a considerable decrease in both peak values was observed, as indicated by low recovery values (Supplementary Materials, Tables S1 and S2). In fact, this instability was also observed at each timepoint, with considerable variation between replicas.

To address the reduced stability of the IS in the samples, sample storage prior to the addition of IS was considered, with the latter being prepared daily and only added to the samples before the derivatization (procedure C). With this approach, sample stability increased in every storage condition, regardless of the considered matrix, with signs of instability only being observed after 96 h of storage, regardless of the storage temperature (Table 4). Considering these results, it is suggested that this method should be applied in the quantification of Lys in biological matrices stored for up to 2 days, and ideally be analyzed within this period of time. Furthermore, samples were found to be more stable than those used in the original method [12], being able of being stored for up 4x more time. In addition, the IS should be prepared and added prior to derivatization, as close as possible to the injection of the samples.

The assay performed to assess applicability, using samples with an unknown Lys concentration achieved concentrations between 112 µM and 127 µM, and rendered an overall recovery value of 104 ± 7%, when comparing the amount of Lys after and before Lys spiking, respectively. These values were very close to 100%, indicating that the method was indeed efficient when quantifying Lys in these samples, demonstrating its potential for future applications.

## 3. Materials and Methods

### 3.1. Materials

Dulbecco’s phosphate buffered saline, Tween^®^ 60, L-lysine monohydrochloride, lithium carbonate, dansyl chloride, metacrilamide, methylamine hydrochloride, triethylamine, and sodium acetate were purchased from Sigma-Aldrich (St. Louis, MO, USA), L-phenylalanine ethyl-ester hydrochloride from Fluka (Fluka Chemie GmbH, Buchs, Switzerland), acetic acid from VWR Chemicals (VWR International S.A.S., Fontenay-sous-Bois, France), and acetonitrile and methanol from Honeywell (Honeywell Riedel-de Häen AG, Seelze, Germany). Aqueous solutions were prepared with double-deionized water (Arium Pro, Sartorius AG, Göttingen, Germany) and all reagents were of analytical grade or higher.

### 3.2. Preparation of the Matrices Samples

Three matrices were considered in this work: Li_2_CO_3_ buffer, PBS buffer, and rumen inoculum. The aqueous Li_2_CO_3_ buffer was 0.04 M and the pH adjusted to 9.5 using acetic acid. The PBS buffer was prepared by diluting Dulbecco’s phosphate buffered saline 10× in water, as described by the manufacturers (pH 7.4). Rumen digesta was freshly collected from the four quadrants of rumen fistulated adult Holstein cows before the morning meal and approximately 1 h prior to the assays. Cows were handled in strict accordance with good animal practice as defined by the national authority and European Union Directive 2010/63/EU. Experimental animal procedures were approved by the Local Animal Ethics Committee of ICBAS-UP, licensed by the Portuguese Directorate-General of Food and Veterinary Medicine of the Ministry for Agriculture and Sea, and conducted by trained scientists (FELASA category C). The rumen digesta was filtered through four layers of cotton gauze [28] and the rumen fluid was diluted at a 1:5 ratio with a buffer solution prepared as a 50:1 mixture of 1) 10 g KH_2_PO_4_, 0.5 g Mg_2_SO_4_·7H_2_O, 0.5 g NaCl, and 0.1 g CaCl_2_·2H_2_O per L of water, and 2) 15 g Na_2_CO_3_ and 1 Na_2_S·9H_2_O per 100 mL of water [29]. 

### 3.3. Derivatization

All matrices were diluted 1:1 in Li_2_CO_3_ buffer (0.04 M, pH 9.5) prior to derivatization. To 50 µL of sample solution were added 50 µL of internal standard (IS) solution (2.3 M L-phenylalanine ethyl-ester prepared in Li_2_CO_3_) and 100 µL of dansyl solution (9.6 M in acetonitrile), homogenized with a vortex and incubated for 30 min at 60 °C in a water bath, protected from the light. After the incubation, 10 µL of methylamine solution (10% in Li_2_CO_3_) were added to consume any excess of derivatizing agent.

### 3.4. Chromatographic Conditions

The chromatographic conditions were adapted from the literature [12]. The HPLC system had two high-pressure pumps (PU-2080 plus), a refrigerated automated injector (AS-2057 Plus), and a fluorescence detector (FP-920) programmed at 330 nm for excitation and 508 nm for emission wavelengths, all from Jasco (Tokyo Japan). The column was a Kinetex^®^ EVO C18 100 Å (100.0 mm × 3.0 mm, 2.6 µm) from Phenomenex (Torrance, CA, USA). The aqueous phase (A) was composed of sodium acetate at 0.02 M with triethylamine at 0.02%, pH set to 4.5 with acetic acid, while the organic phase (B) was a 1:9 mixture of sodium acetate buffer at 0.1 M, pH set to 4.5 with acetic acid, and methanol, respectively. The flow rate was 400 µL per min and the injection volume was 20 µL. The total run length was 22 min under the following gradient—0 min: 47% (A), 13 min: 16% (A), 15 min: 16% (A), 17 min: 47% (A), 22 min: 47% (A). Lysine retention time was 13.5 min, while the IS peak appeared at 15.7 min. 

### 3.5. Method Validation

The chromatographic method was validated for specificity, linearity, accuracy, precision, and range in the three matrices: Li_2_CO_3_ buffer, PBS buffer, and rumen fluid. 

Firstly, blanks (six replicates) of all matrices were analyzed to ensure that no peaks that could overlap with the analytes were present. 

Linearity was evaluated by preparing calibration curves in triplicate on three distinct days (*n* = 9) for all matrices. The curves were constructed by plotting the Lys/IS area ratio vs. the concentration of the seven standard solutions (0, 1, 12.5, 25, 50, 100, and 200 µM), prepared in all matrices under study. Linear regressions were calculated for all the plotted lines, fitted to least squares linear regressions. 

Accuracy and precision were determined by analyzing replicates (*n* = 6) of a CC sample at 25 µM of Lys. Accuracy was determined via recovery, by comparing the concentration of a CC sample after the addition of a known amount of Lys (final concentration of 35 µM) with a CC sample at the same concentration without any additional Lys. To determine precision, CC samples were prepared on the same day and on three different consecutive days, to assess intra-day and inter-day variations, respectively. In the case of accuracy, CC samples with extra-added Lys were compared with CC samples without this addition, after the mathematical subtraction of the added Lys.

The LOD and the LOQ were determined using the signal-to-noise method for all matrices, of 3:1 and 10:1, respectively. 

### 3.6. Sample Stability

Both derivatized and non-derivatized CC samples with 25 µM of Lys (prepared in each matrix with IS addition as previously described) were tested for stability after 24 h, 48 h, and 96 h at RT, 4 °C, and −20 °C. In a second experiment, CC samples with 25 µM of Lys were stored for the same timepoints prior to IS addition, with the latter being prepared and added each day immediately prior to derivatization. In both experiments, and for every condition, six replicates processed were analyzed. Derivatized samples were directly analyzed after the selected time in storage, whereas non-derivatized samples were stored prior to derivatization, which only occurred immediately before they were analyzed. 

### 3.7. Application of the Method towards Lys Quantification

In order to properly assess and validate the method, a different set of samples (aqueous Lys solutions obtained from a previous study [28]) were analyzed. A recovery assay, using six distinct samples of unknown concentrations, was performed by adding a known amount of Lys to the samples, with the total Lys being quantified both before and after this addition. The added Lys (known amount) was subtracted to the Lys determined in the latter, and the resulting value compared with the former.

## 4. Conclusions

In this work, a simple Lys quantification method based in HPLC was optimized for applications in three specific biological matrices. Besides an Li_2_CO_3_ buffer (the standard reactionary medium used in the original method [12]), capable of covering the majority of the more simple matrices, two additional matrices were considered, to account for quantification needs in cellular assays (PBS), and ex vivo or in vivo rumen assays (rumen inoculum), with emphasis for studies regarding dairy cow nutrition, but with the capability of applications in studies where the quantification of Lys, in any of these matrices, is required. This simple, accurate and precise method for Lys quantification was based on chromatographic separation of dansyl derivatives, obtained by pre-column derivatization, followed by fluorescence detection. Furthermore, the method’s total run length was considerably shortened when compared to the original method in which this one was based, to focus specifically on the Lys region, without compromising its separation from sample interferences. The simplicity of the sample preparation approach enables the quantification of a high number of samples simultaneously.

Studies were also performed to determine the most adequate storage temperature to be used when an immediate analysis of samples would not be possible, with a good stability for up to two days being found. This stability was found regardless of the storage temperature, but only when both IS addition and derivatization were performed immediately before analysis. 

The presented results indicate that the developed method could be a great asset for future applications in the field of dairy cow nutrition.

## Figures and Tables

**Table 1:** Chromatographic information of the optimized method.

Compound	tR (min; Mean (RSD))	k	N	α	Rs
Lysine	13.5 (0.2)	6.9	2037	1.2	1.9
IS	15.7 (0.1)	8.2	3275		

α, selectivity factor; IS, internal standard; k, retention factor; N, efficiency (calculated at 25 µM Lys); Rs, resolution; RSD, relative standard deviation; and tR, retention time.

**Table 2:** Regression analysis of calibration curves, detection limit and quantification limit in Li_2_CO_3_ buffer, phosphate buffered saline buffer, and rumen inoculum (*n* = 3).

Matrix	Range (µM)	Slope	Correlation Coefficient	LOD (µM)	LOQ (µM)
Li_2_CO_3_	1–225	0.011265	0.9999	0.12	0.41
PBS	1–225	0.009982	0.9998	0.16	0.53
Rumen	5–225	0.031104	0.9996	1.24	4.14

LOD, limit of detection; LOQ, limit of quantification; and PBS, phosphate buffered saline.

**Table 3:** Determined values of accuracy (*n* = 6) and both intra-day (*n* = 6) and inter-day (*n* = 18) precision for Li_2_CO_3_ buffer, phosphate buffered saline buffer, and rumen inoculum.

Matrix	Concentration (µM)	Accuracy (%)	Precision (%)	
			Intra-Day	Inter-Day
Li_2_CO_3_	25	94 ± 3	2 ± 1	13 ± 7
PBS	25	92 ± 2	2 ± 3	22 ± 10
Rumen	25	92 ± 9	9 ± 5	9 ± 5

PBS, phosphate buffered saline.

**Table 4:** Stability results for both derivatized and non-derivatized samples, at 24 h, 48 h, and 96 h of storage at RT, 4 °C, and −20 °C for Li_2_CO_3_ buffer, PBS buffer, and rumen inoculum, *n* = 5. Values shown as mean ± standard deviation.

Matrix	24 h			48 h			96 h		
	A	B	C	A	B	C	A	B	C
Recovery (%) when stored at RT									
Li_2_CO_3_	99 ± 2	286 ± 83	102 ± 1	75 ± 4	343 ± 67	109 ± 1	76 ± 4	151 ± 762	83 ± 1
PBS	100 ± 1	211 ± 7	100 ± 1	101 ± 1	588 ± 17	105 ± 1	100 ± 1	1976 ± 62	85 ± 1
Rumen	100 ± 3	8414 ± 1472	99 ± 1	107 ± 14	5375 ± 4227	100 ± 1	106 ± 13	2105 ± 2917	95 ± 1
Recovery (%) when stored at 4 °C									
Li_2_CO_3_	101 ± 1	167 ± 5	103 ± 1	115 ± 70	111 ± 3	108 ± 1	344 ± 377	155 ± 6	83 ± 1
PBS	100 ± 1	115 ± 5	100 ± 1	101 ± 1	168 ± 5	105 ± 1	100 ± 1	189 ± 7	85 ± 1
Rumen	99 ± 3	69 ± 2	99 ± 1	101 ± 9	83 ± 21	101 ± 1	99 ± 9	4891 ± 2198	96 ± 1
Recovery (%) when stored at −20 °C									
Li_2_CO_3_	338 ± 111	134 ± 7	102 ± 1	400 ± 98	85 ± 15	108 ± 1	368 ± 96	91 ± 7	83 ± 1
PBS	105 ± 9	105 ± 10	100 ± 1	524 ± 264	122 ± 6	106 ± 1	410 ± 178	110 ± 7	85 ± 1
Rumen	474 ± 91	147 ± 19	99 ± 1	287 ± 62	140 ± 11	101 ± 1	522 ± 422	167 ± 27	96 ± 1

A, samples that were stored after derivatization; B, samples that were stored prior to derivatization; and C, samples that were stored prior to derivatization and IS addition; PBS, phosphate buffered saline; and RT, room temperature.

## Data Availability

No datasets were used or generated during this study.

## Supplementary Materials

Table S1—Variation results for both lysine (Lys) and internal standard (IS) of samples that were stored after derivatization, at 24 h, 48 h, and 96 h of storage at room temperature (RT), 4 °C, and −20 °C for Li_2_CO_3_ buffer, phosphate buffer solution (PBS), and rumen inoculum, *n* = 5. Recovery was determined by dividing the peak area at each time point by the initial peak area (before storage) and the values are shown as mean ± standard deviation. Table S2—Variation results for both lysine (Lys) and internal standard (IS) of samples that were stored prior to derivatization, at 24 h, 48 h, and 96 h of storage at room temperature (RT), 4 °C, and −20 °C for Li_2_CO_3_ buffer, phosphate buffer solution (PBS), and rumen inoculum, *n* = 5. Recovery was determined by dividing the peak area at each time point by the initial peak area (before storage) and the values are shown as mean ± standard deviation.

## Sample Availability

No samples of any compound are available from the authors.

## References

[1] Fraser D.L., Ørskov E.R., Whitelaw F.G., Franklin M.F. (1991). Limiting amino acids in dairy cows given casein as the sole source of protein. Livest. Prod. Sci..

[2] Schwab C.G. (1996). Rumen-protected amino acids for dairy cattle: Progress towards determining lysine and methionine requirements. Anim. Feed Sci. Technol..

[3] Swanepoel N., Robinson P.H., Erasmus L.J. (2010). Amino acid needs of lactating dairy cows: Impact of feeding lysine in a ruminally protected form on productivity of lactating dairy cows. Anim. Feed Sci. Technol..

[4] Schwab C.G., Broderick G.A. (2017). A 100-Year Review: Protein and amino acid nutrition in dairy cows. J. Anim. Sci..

[5] National Research Council (2001). Nutrient Requirements of Dairy Cattle.

[6] Gwatidzo L., Botha B.M., McCrindle R.I. (2013). Determination of amino acid contents of manketti seeds (Schinziophyton rautanenii) by pre-column derivatisation with 6-aminoquinolyl-N-hydroxysuccinimidyl carbamate and RP-HPLC. Food Chem..

[7] Knapp D.R. (1979). Handbook of Analytical Derivatization Reactions.

[8] Lawrence J.F., Frei R.W., Lawrence J.F., Frei R.W. (1976). Chapter 1 Introduction. Journal of Chromatography Library.

[9] Lawrence J.F., Frei R.W., Lawrence J.F., Frei R.W. (1976). Chapter 4 Applications. Journal of Chromatography Library.

[10] Takeuchi T., Molnár-Perl I. (2005). 1.2.5.—HPLC of Amino Acids as Dansyl and Dabsyl Derivatives. Journal of Chromatography Library.

[11] Ribeiro B., Andrade P.B., Silva B.M., Baptista P., Seabra R.M., Valentao P. (2008). Comparative study on free amino acid composition of wild edible mushroom species. J. Agric. Food Chem..

[12] Zhang L., Li Y., Zhou H., Li L., Wang Y., Zhang Y. (2012). Determination of eight amino acids in mice embryonic stem cells by pre-column derivatization HPLC with fluorescence detection. J. Pharm. Biomed. Anal..

[13] Chen G., Li J., Sun Z., Zhang S., Li G., Song C., Suo Y., You J. (2014). Rapid and sensitive ultrasonic-assisted derivatisation microextraction (UDME) technique for bitter taste-free amino acids (FAA) study by HPLC-FLD. Food Chem..

[14] Sanz M.A., Castillo G., Hernandez A. (1996). Isocratic high-performance liquid chromatographic method for quantitative determination of lysine, histidine and tyrosine in foods. J. Chromatogr. A.

[15] Hernandez A., Serrano M.A., Munoz M.M., Castillo G. (2001). Liquid chromatographic determination of the total available free and intrachain lysine in various foods. J. Chromatogr. Sci..

[16] Zhang L., Wang X., Su J., Liu H., Zhang Z., Qin L., He C., Peng L., Guo M., Gao X. (2014). One single amino Acid for estimation the content of total free amino acids in qingkailing injection using high-performance liquid chromatography-diode array detection. J. Anal. Methods Chem..

[17] Riahi S., Ganjali M.R., Hariri M., Abdolahzadeh S., Norouzi P. (2009). Determination of the formation constant for the inclusion complex between Lanthanide ions and Dansyl chloride derivative by fluorescence spectroscopy: Theoretical and experimental investigation. Spectrochim. Acta Part A Mol. Biomol. Spectrosc..

[18] Karabacak M., Cinar M., Kurt M., Poiyamozhi A., Sundaraganesan N. (2014). The spectroscopic (FT-IR, FT-Raman, UV and NMR) first order hyperpolarizability and HOMO–LUMO analysis of dansyl chloride. Spectrochim. Acta Part A Mol. Biomol. Spectrosc..

[19] Zhou S., Zhou Z.-Q., Zhao X.-X., Xiao Y.-H., Xi G., Liu J.-T., Zhao B.-X. (2015). A dansyl based fluorescence chemosensor for Hg2+ and its application in the complicated environment samples. Spectrochim. Acta Part A Mol. Biomol. Spectrosc..

[20] Oldekop M.-L., Herodes K., Rebane R. (2017). Comparison of amino acid derivatization reagents for liquid chromatography atmospheric pressure chemical ionization mass spectrometric analysis of seven amino acids in tea extract. Int. J. Mass Spectrom..

[21] Liu S.-J., Xu J.-J., Ma C.-L., Guo C.-F. (2018). A comparative analysis of derivatization strategies for the determination of biogenic amines in sausage and cheese by HPLC. Food Chem..

[22] Song Y., Xu C., Kuroki H., Liao Y., Tsunoda M. (2018). Recent trends in analytical methods for the determination of amino acids in biological samples. J. Pharm. Biomed. Anal..

[23] Minocha R., Long S. (2004). Simultaneous separation and quantitation of amino acids and polyamines of forest tree tissues and cell cultures within a single high-performance liquid chromatography run using dansyl derivatization. J. Chromatogr. A.

[24] Belenkii B.G., Vilenchik L.Z., Belenkii B.G., Vilenchik L.Z. (1983). Chapter 1 General theory of chromatography. Modern Liquid Chromatography of Macromolecules.

[25] Martins S.M., Wendling T., Goncalves V.M., Sarmento B., Ferreira D.C. (2012). Development and validation of a simple reversed-phase HPLC method for the determination of camptothecin in animal organs following administration in solid lipid nanoparticles. J. Chromatogr. B Anal. Technol. Biomed. Life Sci..

[26] Jajic I., Krstovic S., Glamocic D., Jaksic S., Abramovic B. (2013). Validation of an HPLC method for the determination of amino acids in feed. J. Serb. Chem. Soc..

[27] U.S. Department of Health and Human Services, Food and Drug Administration, Center for Drug Evaluation and Research, Center for Veterinary Medicine (2018). Bioanalytical Method Validation Guidance for Industry.

[28] Albuquerque J., Casal S., Páscoa R.N.M.d.J., Van Dorpe I., Fonseca A.J.M., Cabrita A.R.J., Neves A.R., Reis S. (2020). Applying nanotechnology to increase the rumen protection of amino acids in dairy cows. Sci. Rep..

[29] Marten G.C., Barnes R.F. Prediction of energy digestibility of forages with in vitro rumen fermentation and fungal enzyme systems [ruminants, domesticated birds]. Proceedings of the Workshop on Standardization of Analytical Methodology for Feeds.

