# Supplementary material for: Validation of a Simple HPLC-Based Method for Lysine Quantification for Ruminant Nutrition
Source: Molecules. 2021 Jul 9;26(14):4173. doi: 10.3390/molecules26144173 (PMC8304843; doi:10.3390/molecules26144173)
Supplement: Supplementary file 1 [file molecules-26-04173-s001.zip › molecules-1277680-supplementary.pdf]

*Table S1 – Variation results for both lysine (Lys) and internal standard (IS) of samples that were stored after derivatization, at 24 h, 48 h and 96 h of storage at room temperature (RT), 4 °C and -20 °C for $Li_2CO_3$ buffer, phosphate buffer solution (PBS) and rumen inoculum, n = 5. Recovery was determined by dividing the peak area at each time point by the initial peak area (before storage) and the values are shown as mean ± standard deviation.*

| **Matrix** | **24 h** | | **48 h** | | **96 h** | |
|---|---|---|---|---|---|---|
| | Lys | IS | Lys | IS | Lys | IS |
| **Recovery (%) when stored at RT** | | | | | | |
| **$Li_2CO_3$** | 98 ± 7 | 99 ± 6 | 69 ± 4 | 91 ± 4 | 70 ± 4 | 93 ± 6 |
| **PBS** | 84 ± 2 | 84 ± 2 | 88 ± 4 | 88 ± 4 | 23 ± 8 | 22 ± 8 |
| **Rumen** | 101 ± 5 | 101 ± 6 | 91 ± 8 | 92 ± 8 | 113 ± 6 | 115 ± 6 |
| **Recovery (%) when stored at 4 °C** | | | | | | |
| **$Li_2CO_3$** | 101 ± 5 | 100 ± 5 | 83 ± 12 | 88 ± 26 | 84 ± 16 | 68 ± 40 |
| **PBS** | 84 ± 1 | 87 ± 2 | 83 ± 6 | 83 ± 6 | 87 ± 8 | 87 ± 8 |
| **Rumen** | 99 ± 2 | 100 ± 4 | 93 ± 7 | 92 ± 7 | 105 ± 13 | 78 ± 13 |
| **Recovery (%) when stored at -20 °C** | | | | | | |
| **$Li_2CO_3$** | 51 ± 12 | 21 ± 20 | 37 ± 4 | 10 ± 3 | 38 ± 3 | 11 ± 2 |
| **PBS** | 94 ± 14 | 94 ± 6 | 36 ± 7 | 9 ± 6 | 34 ± 16 | 9 ± 2 |
| **Rumen** | 37 ± 6 | 8 ± 1 | 32 ± 8 | 12 ± 4 | 32 ± 14 | 9 ± 5 |

*Table S2 – Variation results for both lysine (Lys) and internal standard (IS) of samples that were stored prior to derivatization, at 24 h, 48 h and 96 h of storage at room temperature (RT), 4 °C and -20 °C for $Li_2CO_3$ buffer, phosphate buffer solution (PBS) and rumen inoculum, n = 5. Recovery was determined by dividing the peak area at each time point by the initial peak area (before storage) and the values are shown as mean ± standard deviation.*

| **Matrix** | **24 h** | | **48 h** | | **96 h** | |
|---|---|---|---|---|---|---|
| | Lys | IS | Lys | IS | Lys | IS |
| **Recovery (%) when stored at RT** | | | | | | |
| **$Li_2CO_3$** | 227 ± 23 | 56 ± 6 | 80 ± 8 | 17 ± 2 | 144 ± 19 | 10 ± 10 |
| **PBS** | 82 ± 9 | 39 ± 4 | 106 ± 7 | 18 ± 1 | 78 ± 24 | 70 ± 19 |
| **Rumen** | 133 ± 24 | 2 ± 1 | 94 ± 8 | 1 ± 1 | 4 ± 5 | 5 ± 6 |
| **Recovery (%) when stored at 4 °C** | | | | | | |
| **$Li_2CO_3$** | 177 ± 11 | 90 ± 6 | 82 ± 4 | 63 ± 3 | 118 ± 4 | 55 ± 2 |
| **PBS** | 93 ± 5 | 80 ± 3 | 104 ± 7 | 62 ± 4 | 74 ± 7 | 39 ± 4 |
| **Rumen** | 42 ± 4 | 31 ± 4 | 44 ± 3 | 59 ± 4 | 136 ± 31 | 2 ± 1 |
| **Recovery (%) when stored at -20 °C** | | | | | | |
| **$Li_2CO_3$** | 150 ± 14 | 98 ± 7 | 95 ± 22 | 97 ± 6 | 116 ± 16 | 97 ± 7 |
| **PBS** | 96 ± 14 | 96 ± 7 | 111 ± 9 | 91 ± 7 | 78 ± 24 | 70 ± 19 |
| **Rumen** | 137 ± 28 | 94 ± 17 | 145 ± 26 | 103 ± 16 | 160 ± 31 | 97 ± 17 |
